# Reciprocal interactions between osteoclasts and nociceptive sensory neurons in bone cancer pain

**DOI:** 10.1097/PR9.0000000000000867

**Published:** 2021-03-09

**Authors:** Amanda S. Andriessen, Christopher R. Donnelly, Ru-Rong Ji

**Affiliations:** a Department of Anesthesiology, Center for Translational Pain Medicine, Duke University Medical Center, Durham, NC, USA; b Department of Cell Biology, Duke University Medical Center, Durham, NC, USA

**Keywords:** Nociceptor, neuroimmune interactions, osteoclast, bone cancer, cancer pain

## Abstract

Many common cancers such as breast, prostate, and lung cancer metastasize to bones at advanced stages, producing severe pain and functional impairment. At present, the current pharmacotherapies available for bone cancer pain are insufficient to provide safe and efficacious pain relief. In this narrative review, we discuss the mechanisms used by cancer cells within the bone tumor microenvironment (TME) to drive bone cancer pain. In particular, we highlight the reciprocal interactions between tumor cells, bone-resorbing osteoclasts, and pain-sensing sensory neurons (nociceptors), which drive bone cancer pain. We discuss how tumor cells present within the bone TME accelerate osteoclast differentiation (osteoclastogenesis) and alter osteoclast activity and function. Furthermore, we highlight how this perturbed state of osteoclast overactivation contributes to bone cancer pain through (1) direct mechanisms, through their production of pronociceptive factors that act directly on sensory afferents; and (2) by indirect mechanisms, wherein osteoclasts drive bone resorption that weakens tumor-bearing bones and predisposes them to skeletal-related events, thereby driving bone cancer pain and functional impairment. Finally, we discuss some potential therapeutic agents, such as denosumab, bisphosphonates, and nivolumab, and discuss their respective effects on bone cancer pain, osteoclast overactivation, and tumor growth within the bone TME.

## Abstract 2

In this narrative review, we discuss how osteoclasts directly or indirectly interact with pain-sensing sensory neurons (nociceptors) to promote pain in bone cancer.

## 1. Introduction

Cancer is the second leading cause of death in the United States and affects several million people around the world every year.^25^ In addition to being a life-threatening illness in and of itself, cancer frequently presents with many neurological comorbidities such as pain, depression, and anxiety, all of which culminate in substantially reduced quality of life. Approximately 75% of patients with late-stage cancer experience moderate or severe pain,^21,23,31^ and at least half of all patients with metastatic cancer report insufficient pain relief using existing pharmacotherapies.^82^ Despite these startling numbers, the therapeutic options available to treat cancer pain remain limited, with significant limitations in both the efficacy and long-term safety.^31,82^ For example, given the ongoing opioid epidemic, healthcare providers are hesitant to prescribe opioid analgesics due to their potential for addiction, abuse, and misuse, especially as the long-term prognosis for cancer patients is improving.^71^

Breast, prostate, and lung cancers are not only among the most common types of cancer but also have a high likelihood to metastasize to bone, leading to severe bone cancer pain and other comorbidities.^22,84^ In addition, bone metastases are difficult to treat and have poor long-term prognosis.^84,92,93^ The bone marrow presents a favorable site for cancer metastasis due to its slower blood flow and high vascularization.^22,60^ In addition, the bone marrow is regarded as an immunosuppressive tumor microenvironment (TME),^67^ potentially offering a refuge that shields cancer cells from immune surveillance and antitumor immunity. As the bone marrow becomes increasingly infiltrated and populated by locally aggressive cancer cells, they produce mediators that alter the phenotype and function of resident cells that occupy this niche, such as bone-forming osteoblasts (OBs), bone-resorbing osteoclasts (OCLs), and nerve fibers from pain-sensing sensory neurons (nociceptors).^58^ As the bone cancer progresses, it leads to complications referred to as skeletal-related events (SREs), which can include skeletal fractures, spinal cord compression/instability, and systemic hypercalcemia and anemia. SREs themselves are associated with pain, functional impairment, reduced mobility, diminished quality of life, and significantly worse overall survival.^84,92,93^

**Figure 1.**
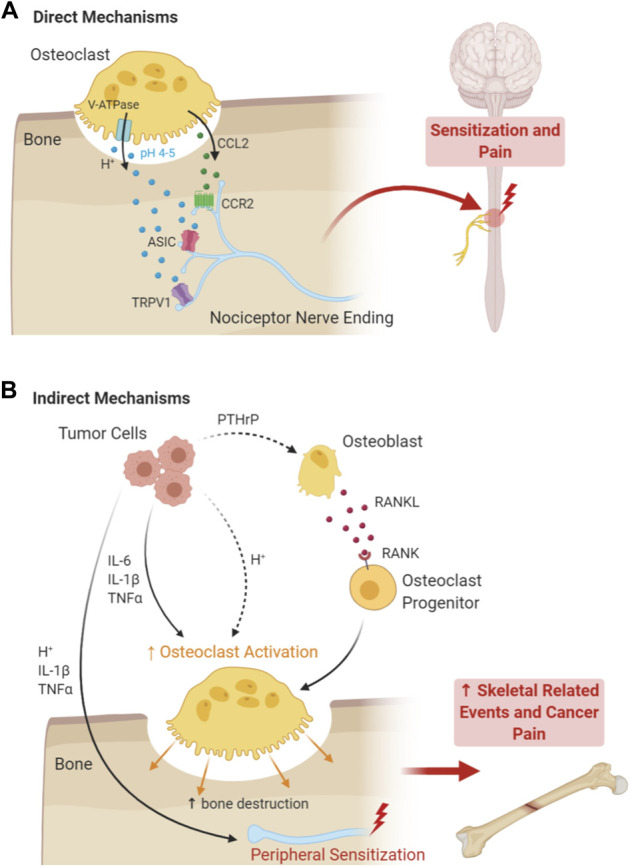
Direct and indirect mechanisms of osteoclasts underlying the pathogenesis of cancer pain. (A) Direct mechanisms by which OCLs produce pain include the induction of local acidosis, mediated by the action of V-ATPase, which activates acid-sensing receptors on peripheral nociceptor terminals in the bone tumor microenvironment, including TRPV1, ASIC1b, and ASIC3. OCLs also release chemokines, such as CCL2, at the absorption lacuna, which activate CCR2 receptors on local nociceptor terminals. OCLs' sustained activation of nociceptors leads to peripheral sensitization, central sensitization, spinal glia activation, and severe pain. In addition to factors released by osteoclasts, tumor cells release a host of factors that contribute directly to nociceptor sensitization and nerve sprouting, including cytokines (IL-1β and tumor necrosis factor), VEGF ligands, NGF, G-CSF, and GM-CSG (not shown). Tumor cells also increase the acidity of the extracellular environment, further activating proton-sensing nociceptors. (B) Indirect mechanisms by which OCLs produce bone cancer pain include tumor-induced induction of osteoclastogenesis, osteoclast hypertrophy, and accelerated osteoclast absorption of bone. This leads to decreased bone mass, enabling further tumor invasion of the bone tissue, and increased likelihood for skeletal-related events such as bone fracture and nerve compression, leading indirectly to bone cancer-related pain. NGF, nerve growth factor; VEGF, vascular endothelial growth factor.

Importantly, bones are extensively innervated by afferent fibers from sensory neurons whose cell bodies are located in the dorsal root ganglion (DRG) or trigeminal ganglion, which extend central projections to the spinal cord or brainstem to relay this sensory information to the central nervous system. In particular, DRG nociceptors densely innervate both the external and internal surfaces of long bones, where they are poised to detect potentially hazardous stimuli such as fractures or neoplasms. Bone cancer pain is frequently described as a polymodal pain condition, presenting somewhat uniquely with aspects of inflammatory, neuropathic, compression, and ischemic pain, and for that reason, it is frequently regarded as its own entity.^31^ In this narrative review, we discuss the molecular mechanisms that cancer cells, osteoclasts, and sensory nerve fibers use to interact with one another within the bone marrow TME to drive bone cancer pain. In particular, we highlight (1) how tumor cells perturb and hijack the bone marrow microenvironment and (2) the mechanisms that osteoclasts use to drive bone cancer pain through direct mechanisms (eg, production of pronociceptive mediators that act on nearby sensory nerve fibers themselves) and indirect mechanisms (eg, bone resorption leading to increased SREs; Fig. 1). We conclude with a discussion of potential therapeutic targets for bone cancer pain.

## 2. Osteoclasts: specialized bone-resident phagocytes with many functions

OCLs are specialized myeloid-derived phagocytes that reside on the surface of bones where they are responsible for bone resorption, aiding in homeostatic bone turnover under steady-state conditions.^94^ OCLs are large multinucleated cells that may reach a diameter of >100 µm. OCLs differentiate from cells of the monocyte/macrophage lineage through a process that is tightly regulated by macrophage-colony stimulating factor (M-CSF) and receptor activator of nuclear factor kappa-β ligand (RANKL) signaling.^7,54,64^ Receptor activator of nuclear factor kappa-β ligand and M-CSF are produced by nearby stromal cells and OBs and are necessary for osteoclastogenesis, driving the upregulation of osteoclast-specific genes and fusion of precursor cells to form mature, multinucleated osteoclasts.^7,87^ Even once formed, RANKL signaling promotes continued survival and activation states of mature osteoclasts and hence is an important target for regulating osteoclast activity.^7,39,87^ Skeletal maintenance and homeostasis under steady-state conditions requires a tight balance between OB-mediated bone formation and OCL-mediated bone resorption. Pathological overactivation or underactivation of either component can lead to bone pathologies.^32^ To perform bone resorption, OCLs create a local acidic and lytic environment at the bone–OCL interface within resorption lacuna (Howship lacunae), leading to breakdown of the bone mineral structure.^59^ The vacuolar-ATPase (V-ATPase) proton pump on the osteoclast border along with a chloride ion/HCO_3_ exchanger creates a proton and hydrochloric acid-rich microenvironment to dissolve the inorganic bone matrix. Lysosomal enzymes such as acid hydrolases released by the osteoclast into the lacuna break down the collagen fibers and OCLs phagocytose the degraded materials.^13^

Given their origin within the monocyte/macrophage lineage of innate immune cells, it is perhaps unsurprising that OCLs are now recognized to be highly plastic cells that play important roles in immunosuppression or immunoactivation, depending on the cellular context. Under steady-state conditions, OCLs produce immunosuppressive cytokines such as interleukin-10 (IL-10) and transforming growth factor-β, which induce immunosuppressive regulatory T-cells (Treg), which also inhibit OCL differentiation. However, like macrophages, osteoclasts possess the ability to sense and respond to infection, tissue damage, and local inflammation, possessing similar phagocytic and antigen-presenting capabilities. Within this capacity, pathological inflammation drives macrophages to produce proinflammatory cytokines such as IL-1β, IL-6, and tumor necrosis factor, both through direct secretion and indirectly through activation of proinflammatory T cells.^26,49,54,62^ Thus, OCLs are uniquely positioned, both physically and functionally, to participate in both bone resorption and immunoregulation depending on the status of the surrounding microenvironment, and they use both of these capabilities concurrently in many diseases of the bone, including bone cancer.^59^

## 3. Cancer cells drive osteoclast overactivation, leading to “the vicious cycle” of bone destruction

Many studies have established that osteoclasts play a key role in bone cancer progression and bone cancer pain. Under steady-state conditions, bone is turned over through a balanced cycle of bone resorption and bone formation through the reciprocal actions of OCLs and OBs, respectively. However, as tumor cells populate the bone marrow microenvironment, they deregulate the normal bone remodeling cycle, pushing the balance towards osteoclast-mediated bone resorption, thereby facilitating further cancer cell invasion of the bone tissue, a model that is termed the “vicious cycle.” ^1,59,75^ Osteolytic tumor (eg, those that promote bone destruction) cells increase osteoclast activity through secretion of proinflammatory cytokines such as IL-1β, IL-6, and TNF, and chemokines such as chemokine C-C motif ligand 2 (CCL2), which act directly on osteoclasts. In addition, tumor cells increase osteoclastogenesis through secretion of parathyroid hormone-related protein, a paracrine regulator of osteoclastogenesis that induces RANKL secretion, a critical regulator of osteoclast differentiation.^10,50,61,89,98^ In fact, osteolytic cancer cells engage in osteomimicry in many respects, mimicking OBs, the normal positive regulator of OCLs under homeostatic conditions.^4,45,68^ In return, osteoclasts, both increased in numbers and in activation, degrade the bone matrix and facilitate local invasion of cancer cells into the bone tissue while concurrently releasing growth factors, chemokines, and cytokines that also promote tumor growth, thus completing “the vicious cycle.” ^4,10,59^ Thus, osteolytic cancer cells drive a self-serving cycle of osteoclast proliferation and hypertrophy at the bone-tumor interface, leading to accelerated bone destruction.^43,59^ This accelerated bone destruction leads to decreased bone mass and increased fragility, increasing the likelihood of subsequent nerve compression and SREs such as fractures, thereby indirectly producing bone cancer pain,^44,97^ as opposed to direct engagement of nociceptive nerve fibers by osteoclasts through the production of pronociceptive mediators (Fig. 1).^1,22,44,56,97^

Dysregulation of OCLs is also observed in osteoblastic tumor types (eg, those involving ectopic or excessive bone formation). In contrast to osteolytic cancers, osteoblastic cancer cells increase OB activity and decrease OCL formation and/or activity.^90^ Endothelin-1 (ET-1), a 21-amino acid peptide released from endothelial cells under steady-state conditions and hypersecreted by tumor cells, is a known regulator of OB function and a potent contributor to cancer-induced nociception.^33,66,99^ In cocultures of human osteoblastic prostate cancer cells with bone slices, osteoclastic bone resorption is significantly attenuated, an effect which could be rescued after application of an ET-1 neutralizing antibody. ET-1 was also shown to regulate osteoclast motility and bone resorption in a concentration-dependent manner.^2,19,96^ Thus, in primarily osteoblastic cancers, such as metastatic prostate cancer,^90^ ET-1 may negatively regulate osteoclast function while directly contributing to cancer-induced nociceptor sensitization.

## 4. Osteoclasts produce pronociceptive mediators to drive bone cancer pain

### 4.1. Extracellular acidity as an activator of nociceptors

In addition to producing bone cancer pain indirectly through accelerated bone destruction and increased risk of painful SREs, osteoclasts also directly engage nerve fibers from nociceptive sensory neurons to produce pain through multiple mechanisms. Both overactivation of osteoclasts and local tumor growth creates a highly acidic extracellular TME that extends far beyond the resorbing lacunae, activating acid-sensing channels such as those present on the local peripheral terminals of nociceptors, such as transient receptor potential channel-vanilloid subfamily-1 (TRPV1) and acid-sensing ion channels (ASICs), including ASIC1a, 1b, and 3^1,31,53,97^ (Fig. 1). In mice, inhibiting the osteoclast proton pump (V-ATPase) with an inhibitor of H^+^ secretion, bafilomycin A1, reduced pain behaviors in mice inoculated with intratibial multiple myeloma cells or Lewis lung cancer (LLC) cells, suggesting that the V-ATPase contributes to bone cancer pain.^36,88^ Moreover, mice lacking TRPV1, a nonselective cation channel expressed in nociceptive neurons that can be activated by heat, capsaicin, and protons,^80^ exhibited reduced LLC-induced bone cancer pain compared with WT mice.^88^

Acid-sensing ion channels are neuronal proton-gated cation channels that are also proposed to be responsible for detecting extracellular acidosis in bone cancer states.^101^ ASIC1a and ASIC2 subunits are primarily expressed in the central nervous system, whereas ASIC1b and ASIC3 are highly expressed by peripheral sensory neurons, including nociceptors.^97^ ASIC3 is activated in bone cancer and found in peripheral nociceptive nerve fibers in the bone TME.^3,97^ Interestingly, mRNA and protein expression for ASIC1a, 1b, and 3 subunits were all upregulated in rodent bone cancer models,^63,97,104^ suggesting possible role in bone cancer pain. Interestingly, a selective ASIC3 antagonist, APETx2, was able to markedly reduce bone pain in a murine intratibial multiple myeloma model and decreased excitability of DRG neurons cocultured with human multiple myeloma cells,^36^ providing functional evidence for the role of ASIC3.

### 4.2. Nociceptor sensitization in bone cancer pain

Osteoclasts also release chemokines such as CCL2, which activates C-C chemokine receptor type 2 (CCR2) receptors on local peripheral nociceptor terminals (Fig. 1).^89^ In addition, tumor cells also release a variety of proinflammatory cytokines such as IL-1β and tumor necrosis factor, which also act on their receptors that are present on peripheral nociceptor terminals.^5^ The continual activation of nociceptor afferents present within the bone TME causes spontaneous pain and leads to sensitization of both peripheral and central nociceptors (termed peripheral sensitization and central sensitization), leading to increased sensitivity to sensory stimuli and hallmarks of neuropathic pain such as mechanical allodynia (eg, pain evoked by a normally innocuous stimulus such as light touch).^24,46,56^ Implantation of fibrosarcoma cells into and around the calcaneus bone in mice leads to the development of spontaneous activity in a subset of C-nociceptors and increased firing in response to heat stimulus at early stages.^11^ Notably, this study also demonstrated a time-dependent alteration in nerve fiber sprouting, with increased epidermal nerve fiber density in the skin overlying the implanted tumor at early stages, but a sharp loss of epidermal nerve fibers in later stages, indicative of neuropathy.^11^

ET-1 is present in many human cancer types that have a high incidence of metastasis to bone, including prostate, lung, and breast cancers, and has also been demonstrated to contribute to C-nociceptor sensitization. ET-1 is upregulated in animal models of cancer-induced hyperalgesia and allodynia,^65,72,86^ and antagonists selective for the ET-1 receptor, ET_A_, were shown to attenuate cancer pain in mice^66,72,86^ and humans.^14^ ET-1 has also been shown to contribute to peripheral sensitization in murine bone cancer models. In mice implanted with fibrosarcoma cells into and around the calcaneus bone, ET-1 injected into the receptive fields of C-nociceptors innervating the hind paw evoked an increase firing rate in control and tumor-bearing mice, whereas application of the ET_A_ receptor antagonist BQ-123 attenuated tumor-evoked spontaneous activity and sensitization to heat in C-nociceptors of tumor-bearing mice.^33^ Thus, ET-1 seems to contribute to peripheral sensitization in a variety of cancers, including bone cancer. Notably, activation of ET_B_ receptor was also shown to produce local analgesic effect.^47^

Osteolytic tumor cells have also been shown to release ligands of vascular endothelial growth factor (VEGF) receptor 1 (VEGFR1), including VEGF-A, VEGF-B, and PLGF-2. Interestingly, intraosseous implantation of osteolytic sarcoma cells in mice produced robust pain, peripheral nerve remodeling, and nociceptor sensitization, including increased TRPV1 expression in distal branches of the sciatic nerve. Notably, this was due to cancer cell release of VEGF ligands binding to VEGFR1 on sensory afferent fibers, as sensory neuron-specific deletion of VEGFR1 and pharmacological blockade of VEGFR1/VEGF signaling suppressed cancer pain and attenuated peripheral nerve sprouting into the tumor stroma.^74^ In addition, bone cancer cells also sensitize nerve fibers and induce peripheral sprouting through the release of granulocyte-colony-stimulating factor (G-CSF) and granulocyte-macrophage colony-stimulating factor (GM-CSF), whose receptors are present on local peripheral nociceptor afferents. Granulocyte-colony-stimulating factor and GM-CSF activation of their receptors led to peripheral sensitization and pain behaviors, as well as increased nerve sprouting and accelerated tumor growth. In mice, loss of this signaling axis through pharmacologic inhibition of G-CSF or GM-CSF signaling led to reduced bone cancer pain, reduced peripheral nerve sprouting, and attenuated tumor growth. Furthermore, sensory neuron-selective knockdown of GM-CSFR using RNA interference attenuated bone cancer pain and intratumoral nerve sprouting, providing direct evidence of a tumor-nerve signaling mechanism.^73^ Notably, whether this also altered osteoclast numbers, osteoclast activity, or bone destruction was not tested, but it is likely that G-CSF or GM-CSF may also alter osteoclastogenesis, as has been demonstrated in a noncancer model in mice.^48^ In addition to understanding nerve sprouting in the context of cancer pain, it is noteworthy that nociceptive sensory neurons have been shown to contribute to tumor growth and progression in a variety of preclinical cancer models.^55,69,70,103^ Thus, nerve sprouting has important implications for both cancer pain and cancer progression. Cancer-induced sensory nerve sprouting has been observed in a variety of preclinical models,^6,41,42,51^ and is controlled by a variety of factors that also contribute to peripheral sensitization and pain, including bradykinins, endothelins, and growth factors.^42,57^ In particular, nerve growth factor (NGF) released from macrophages and cancer cells seems to play a critical role in nerve sprouting, engaging its receptor tropomyosin receptor kinase A, which is present on sympathetic and nociceptive sensory nerve fibers.^41,42^ In a mouse model of prostate cancer-induced bone pain, administration of an anti-NGF neutralizing antibody preemptively (eg, before nerve sprouting) or at late stages could attenuate nerve sprouting and cancer pain.^42^ Phase 2 clinical trials with intravenous administration of tanezumab, an anti-NGF monoclonal antibody, provided significantly improved pain scores over a 16-week period in patients with metastatic cancer concurrently taking daily opioids, validating efforts aimed at further development of anti-NGF therapies.^77^

Interestingly, central sensitization resulting from bone cancer in the periphery also leads to activation of spinal microglia and astrocytes, which sustain central sensitization and augment bone cancer pain pathogenesis,^91,95,102^ as well as chronic pain in a variety of other injury conditions.^17,40^ In a rat model of CIPB in which MRMT-1 mammary tumor cells are administered through intratibial injection, the receptive field size and ratio of wide dynamic range (WDR) and nociceptive-specific neurons was significantly increased, a change that reflects local plasticity indicative of central sensitization. ^81^ Tumor-bearing animals exhibited an increased number of WDR neurons in the superficial dorsal horn (SDH), increasing the probability of response to low-threshold peripheral inputs, likely contributing to the clinical features of allodynia.^81^ In rats, the emergence of cancer-induced behavioral hypersensitivity closely parallels altered plasticity in the superficial SDH.^27,95^ Interestingly, sustained treatment with gabapentin, a potent modulator of spinal calcium channel activity leading to decreased neurotransmitter release from afferents, was shown to reverse these pathological changes in plasticity, suggesting that gabapentin may be of therapeutic value for cancer-induced bone pain.^28^ In theory, therapeutics targeting osteoclasts, nociceptors, cancer cells, or any other cell type that leads to reduced input from peripheral afferents is likely to aid in attenuating the pathological changes that occur in central sensitization, such as glial cell activation and neural plasticity in the superficial SDH. In mice, intrafemoral injection of 2472 osteolytic sarcoma cells induced robust bone destruction, spontaneous pain, and astrocyte activation. Interestingly, administration of osteoprotegerin (OPG), a soluble decoy receptor of RANKL, sharply reduced osteoclastogenesis, bone destruction, cancer-induced pain behaviors, and spinal astrocyte activation.^37^ This study also nicely demonstrates the difficulty in parsing out the relative contributions of tumor cells, osteoclasts, and activated spinal glial cells to nociceptor activation and pain in bone cancer because each of these components are intricately linked and it is difficult to alter one without reciprocally influencing the others.

Other cells within the myeloid cell lineage such as microglia and macrophages have been demonstrated in preclinical studies to contribute to pain pathogenesis, but seem to do so in a sexually dimorphic manner (eg, differently in males and females).^17,18,52,78^ For example, intrathecal (eg, spinal) administration of microglial inhibitors such as minocycline and propentofylline, or microglial ablation using MAC-1-saporin toxin, is sufficient to attenuate nerve injury-induced pain in male but not female mice.^78^ In the absence of microglia, female mice use a mechanism driven by adaptive immune cells.^78^ Sex dimorphism may be dependent on the pathological pain condition in question, however, as microglia have been found to contribute to bone cancer pain. In female rats, intratibial inoculation with Walker 256 mammary gland carcinoma cells led to robust mechanical and thermal hypersensitivity as well as spontaneous pain, which was accompanied by robust activation of spinal microglia. In this model, inhibition of spinal microglia with intrathecal minocycline significantly attenuated mechanical and thermal hypersensitivity, suggesting microglia may contribute to bone cancer pain in both males and females in this model.^95^ Similarly, macrophages seem to contribute to the genesis of pathological pain in several models,^18^ but may do so using distinct signaling mechanisms in males and females.^52^ At present, sexual dimorphism in the contribution of osteoclasts to cancer pain has not been demonstrated, nor have male- or female-specific pronociceptive osteoclast signaling mechanisms been identified. Thus, it will be interesting to follow this line of research to see whether osteoclasts, like microglia and macrophages, contribute to bone cancer pain or other chronic pain conditions in a sexually dimorphic manner.

## 5. Therapeutic targets for bone cancer pain: effects on cancer pain, bone destruction, and tumor growth

### 5.1. Food and drug administration (FDA) FDA-approved treatments for bone cancer pain

A limited number of therapeutic strategies directly targeting osteoclasts have been developed for primary or metastatic bone cancer to limit bone destruction and attenuate bone cancer pain (Table 1). Bisphosphonates such as pamidronate, clodronate, and zoledronic acid are the clinical gold-standard treatment for metastatic bone cancer pain.^22,29^ Bisphosphonates are selectively taken up by activated osteoclasts and interfere in cellular metabolism, leading to osteoclast apoptosis and a subsequent reduction in osteoclast-mediated bone resorption and tissue acidosis.^29^ Thus, when effective, bisphosphonates reduce both bone cancer-induced SREs and pain, but efficacy is highly variable across tumor types,^85^ with only 50% of patients showing reduced SREs. In addition, bisphosphonates exhibit minimal antitumor properties, and 30% to 50% of patients taking bisphosphonates develop further metastases.^92,100^ There are also serious complications associated with long-term use of bisphosphonates, including renal insufficiency and bisphosphonate-related osteonecrosis of the jaw, and hence, it is recommended that they are not used for more than 2 years.^83,100^

**Table 1:** Therapeutic strategies to treat primary or metastatic bone cancer pain.

Treatment	Model	Mechanism of action	Effects on nociception/pain	Effects on osteoclast numbers/activity	Effects on tumor growth	Reference
**FDA-approved treatments**						
Bisphosphonates (clodronate, pamidronate, and zoledronic acid)	Bone cancer patients	Absorbed by mature OCL, induce OCL apoptosis	↓	↓	—	22,29
RANKL inhibition (denosumab and osteoprotegerin)	Bone cancer patients	Blocks osteoclastogenesis through inhibition of RANKL signaling	↓	↓	—	15,20,35,76
**Prospective treatments**						
TRPV1 antagonist (resiniferatoxin)	Murine and canine models	Ablation of TRPV1-expressing neurons and/or their projections	↓	—	—	8,9,34
ASIC3 antagonist (APETx2)	Mouse multiple myeloma model	Inhibits ASIC3 channels on peripheral nociceptor terminals	↓	—	—	36
Anti-PD-1 therapy (nivolumab)	Murine bone cancer model (LLC)	Inhibits PD1-mediated osteoclastogenesis; promotes antitumor immunity; and decreases CCL2 secretion	↓	↓	↓	89
Anti-NGF therapy	Murine bone cancer model and phase 2 clinical trials	Inhibits NGF released from cancer cells and macrophages, and reduces nerve sprouting	↓	-/?	—	42,77
CSFR1 inhibitor (pexidartinib)	Murine and rat cancer-induced bone pain models	Multitargeted tyrosine kinase inhibitor that inhibits phosphorylation of CSF1R	↓	↓	↓	79

ASIC, acid-sensing ion channel; NGF, nerve growth factor; RANKL, receptor activator of nuclear factor kappa-β ligand; TRPV1, transient receptor potential channel-vanilloid subfamily-1.

Beyond bisphosphonates, denosumab is currently the only other clinically approved biological treatment targeting osteoclasts for bone cancer metastases and bone cancer pain.^100^ Denosumab is a monoclonal antibody that works by inhibiting RANKL signaling that is required for osteoclastogenesis and maintenance of osteoclast activity, thereby interrupting osteoclastogenesis and subsequent bone resorption.^35^ In reviews of phase III clinical trials of patients with bone metastases, denosumab delayed the onset of SREs and development of moderate/severe pain by about 1 month in comparison with zoledronic acid (regarded as the most effective bisphosphonate).^20,76^ These effects were paired with increased quality of life and decreased use of opioids for pain management. Because denosumab has been established to have better outcomes compared with bisphosphonates in pain and SREs, it will likely continue to gain popularity as an osteoclast-targeting therapeutic for metastatic bone cancer.

### 5.2. Prospective bone cancer pain therapeutics emerging from preclinical studies

In addition to the currently used therapeutics with demonstrated clinical efficacy, several promising approaches to control bone cancer pain and/or SREs have emerged from preclinical studies (Table 1). As a step towards providing palliative care (eg, pain relief) for patients with poor long-term prognosis, some prospective therapeutics targets have focused on targeting nociceptors. For example, in canines with bone cancer, intrathecal administration of the TRPV1 super agonist resiniferatoxin (RTX) significantly reduced pain, lameness, and required treatment unblinding and/or adjustment of analgesic protocol significantly later than control animals.^8,9^ Intra-articular injection of RTX has also shown efficacy in reducing pain and functional impairment in canines with osteoarthritis, providing a potential rational for administration into the bone TME in canines with bone cancer.^38^ Although these studies focused on cancer pain and functional impairment as their endpoint, nociceptors have been shown to contribute to tumor progression in many preclinical models,^55,69,70,103^ and thus, it is possible that RTX or other nociceptor ablation approaches could also confer antitumor properties. However, at present, it is unknown whether RTX alters tumor progression or osteoclast resorption, so its effectiveness must be taken in the context of palliative care. In addition to ablative strategies, selective strategies targeting targets on peripheral terminals within the bone TME have also been proposed. APETx2, a selective antagonist for ASIC3 receptor, reduced bone pain in a murine model of multiple myeloma bone pain, warranting further investigation.^36^

In the presence of a pro-osteoclastogenic microenvironment, osteoclasts have also been demonstrated to upregulate the checkpoint inhibitory molecule programmed cell death ligand-1 (PD-L1), which signals through its receptor programmed cell death protein-1 (PD-1). Notably, PD-L1/PD-1 signaling promotes an immunosuppressive environment, dampening activation of T-cell subsets required for antitumor immune responses. Thus, therapies targeting checkpoint inhibitory pathways such as the PD-L1/PD-1 signaling axis have emerged as a gold-standard cancer immunotherapy treatment of choice.^30,54^ Recently, anti-PD-1 therapy with the clinically used monoclonal antibody nivolumab was demonstrated to reduce bone cancer pain and osteoclast formation in a murine model of bone cancer.^89^ In addition, mice lacking PD-1 exhibited reduced bone cancer pain after intrafemoral inoculation with LLC cells. Interestingly, *Pd1*^*−/−*^ mice also exhibit pain hypersensitivity and reduced osteoclast numbers at baseline.^16,89^ Within the bone TME, PD-L1 promoted RANKL-induced osteoclastogenesis through JNK activation and CCL2 secretion. In addition, CCL2 directly activated CCR2-expressing DRG nociceptors, as evidenced by attenuated bone cancer pain after systemic administration of the CCR2 antagonist RS504393.^89^ Given that anti-PD-1 therapies are already used clinically in patients with various cancers, this may be an attractive new therapeutic option for patients with primary or metastatic bone cancer that could aid in pain control and bone destruction.

Along the same lines of denosumab in targeting regulatory molecules of OCLs, inhibitors of CSF1 and its receptors have also shown therapeutic potential. CSF1R inhibitors such as pexidartinib (PX3397) have shown clinical efficacy in several clinical trials of soft tissue cancers and have been shown to reduce pain in mouse and rat cancer-induced bone pain models. In the murine and rat prostate cancer-induced bone pain model, there was a marked reduction in pain as well as reduced tumor growth, reduced formation of new tumor colonies, and attenuated tumor-induced bone resorption by osteoclasts.^79^ Pexidartinib has advanced to phase 2 clinical trials in patients with metastatic prostate cancer in bone, but these trials have yet to yield results.^12^ Further research on the therapeutic potential of CSFR1 inhibitors for primary or metastatic bone cancer pain is warranted, given the positive results in preclinical and early clinical trials thus far.

## 5. Conclusions

As cancer therapies continue to prolong lifespan, developing increasingly safe and effective long-term therapies to treat cancer pain becomes of paramount importance. Even for patients with poor long-term prognosis, the ability to provide palliative care to patients suffering from intensely painful bone cancer pain is critical to improve their quality of life. Osteoclasts are critical players in the development and progression of bone cancer pain through indirect mechanisms involving bone destruction and SREs, and direct mechanisms involving nociceptor activation by inflammatory mediators and extracellular acidosis. In addition to contributing to bone cancer pain pathogenesis, osteoclast overactivation also promotes tumor growth. Thus, targeting osteoclasts in patients with bone cancer has the potential to provide synergistic pain relief while concurrently slowing tumor progression, and a number of therapeutics show promise. Bisphosphonates and denosumab are 2 common biologics that are approved to target osteoclasts in the context of bone cancer, but even these therapies are restricted to palliative care. Other potential therapeutics, such as resiniferatoxin and APETx2, target nociceptors directly and have started moving to phase I clinical trials or have shown efficacy in animal models. Finally, PD-1 antagonists such as nivolumab and CSFR1 inhibitors such as pexidartinib are relatively new to the field of bone cancer pain but show promising results in preclinical models that warrant further investigation. The ideal agent is one that can provide multifaceted pain relief, inhibition of osteoclast overactivation, and possesses antitumor properties, and further studies aimed at understanding reciprocal interactions between cancer cells, osteoclasts, and nociceptors are likely to generate new therapeutic targets and mechanistic insights.

## Disclosures

R.R. Ji is a consultant of Boston Scientific and received research support from the company. These activities are not related to this review. The remaining authors have no conflicts of interest to disclose.

C.R. Donnelly received support from the John J. Bonica Trainee Fellowship and NIH T32 GM008600. Illustrations were created by A.S. Andriessen and C.R. Donnelly using BioRender.
